# Antidiabetic Activity of Elephant Grass (Cenchrus Purpureus (Schumach.) Morrone) *via* Activation of PI3K/AkT Signaling Pathway, Oxidative Stress Inhibition, and Apoptosis in Wistar Rats

**DOI:** 10.3389/fphar.2022.845196

**Published:** 2022-03-02

**Authors:** Oluwafemi Adeleke Ojo, Abosede Itunuoluwa Oni, Susan Grant, Jennifer Amanze, Adebola Busola Ojo, Odunayo Anthonia Taiwo, Rotdelmwa Filibus Maimako, Ikponmwosa Owen Evbuomwan, Matthew Iyobhebhe, Charles Obiora Nwonuma, Omorefosa Osemwegie, Anthonia Oluyemi Agboola, Christopher Akintayo, Nnaemeka Tobechukwu Asogwa, Nada H. Aljarba, Saad Alkahtani, Gomaa Mostafa-Hedeab, Gaber El-Saber Batiha, Oluyomi Stephen Adeyemi

**Affiliations:** ^1^ Department of Biochemistry, Landmark University, Omu-Aran, Nigeria; ^2^ Department of Biochemistry, Ekiti State University, Ado-Ekiti, Nigeria; ^3^ Department of Biochemistry, Chrisland University, Abeokuta, Nigeria; ^4^ Department of Microbiology, Landmark University, Omu-Aran, Nigeria; ^5^ Department of Biochemistry, Wesley University, Ondo, Nigeria; ^6^ Department of Physiology, Afe Babalola University, Ado-Ekiti, Nigeria; ^7^ Central Research Laboratory, University Road, Tanke Ilorin, Nigeria; ^8^ Department of Biology, College of Science, Princess Nourah Bint Abdulrahman University, Riyadh, Saudi Arabia; ^9^ Department of Zoology, College of Science, King Saud University, Riyadh, Saudi Arabia; ^10^ Department of Pharmacology and Health Research Unit, Medical College, Jouf University, Al-Jawf, Saudi Arabia; ^11^ Department of Pharmacology, Faculty of Medicine, Beni-Suef University, Beni Suef, Egypt; ^12^ Department of Pharmacology and Therapeutics, Faculty of Veterinary Medicine, Damanhour University, Damanhour, Egypt

**Keywords:** diabetes, inflammation, apoptosis, PI3K/akt signalling pathway, aqueous extract of Cenchrus purpureus

## Abstract

**Ethnopharmacological Relevance:** The management of diabetes over the years has involved the use of herbal plants, which are now attracting interest. We assessed the antidiabetic properties of aqueous extract of *C. purpureus* shoots (AECPS) and the mechanism of action on pancreatic *ß*-cell dysfunction.

**Methods:** This study was conducted using Thirty-six 36) male Wistar rats. The animals were divided into six equal groups (*n* = 6) and treatment was performed over 14 days. To induce diabetes in the rats, a single dose of 65 mg/kg body weight of alloxan was administered intraperitoneal along with 5% glucose. HPLC analysis was carried out to identified potential compounds in the extract. *In vitro* tests α-amylase, and α-glucosidase were analyzed. Body weight and fasting blood glucose (FBG) were measured. Biochemical parameters, such as serum insulin, liver glycogen, hexokinase, glucose-6-phosphate (G6P), fructose-1,6-bisphosphatase (F-1,6-BP), interleukin-6 (IL-6), tumor necrosis factor-alpha (TNF-α), and nuclear factor kappa B (NF-ĸB), were analyzed. Additionally, mRNA expressions of phosphatidylinositol 3-kinase/protein kinase B (PI3K/AKT), B-cell lymphoma 2 (Bcl-2), and proliferating cell nuclear antigen (PCNA) were each evaluated.

**Results:** This *in vitro* study showed inhibitory potency of *Cenchrus purpureus* extract (AECPS) as compared with the positive controls. AECPS showed a gradual decrease in alloxan-induced increases in FBG, total cholesterol (TC), triglycerides (TG), low density lipoprotein (LDL-c), G6P, F-1,6-BP, malondialdehyde (MDA), IL-6, TNF-α, and NF-ĸB and increased alloxan-induced decreases in liver glycogen, hexokinase, and high density lipoprotein (HDL-c). The diabetic control group exhibited pancreatic dysfunction as evidenced by the reduction in serum insulin, homeostasis model assessment of *ß*-cell function (HOMA-*β*), expressions of PI3K/AKT, Bcl-2, and PCNA combined with an elevation in homeostatic model assessment of insulin resistance (HOMA-IR). High performance liquid chromatography (HPLC) revealed 3-O-rutinoside, ellagic acid, catechin, rutin, and kaempferol in AECPS.

**Conclusion:** AECPS showed efficient ameliorative actions against alloxan-induced pancreatic dysfunction, oxidative stress suppression as well as, inflammation, and apoptosis via the activation of PI3K/AKT signaling pathways.

## 1 Introduction

Pancreatic beta cells are groups of endocrine cells responsible for the synthesis, storage, and release of insulin. Insulin is a hormone that counteracts antagonizes hyperglycemic hormones, like glucagon, glucocorticosteroids, and epinephrine, to keep circulating glucose concentrations within a narrow physiologic range (Marchetti et al., 2017). Damage to beta cells can lead to hyperglycemia (diabetes mellitus) a common metabolic disorder of carbohydrates, fats, and proteins that is quickly becoming an epidemic (Hudish et al., 2019). Statistics indicate that 9.3% of the world’s population in 2019 is presently living with diabetes; this figure is expected to increase to about 10.9% by 2045 (Saeedi et al., 2019). The disease’s high prevalence, progressive nature, diverse pathogenesis, and complications necessitate rapid treatment options (Osadebe et al., 2014). A variety of treatments, such as insulin therapy, pharmacotherapy, and diet therapy, are currently available for controlling this disease.

Glucose-lowering drugs have anti-diabetic effects through a variety of ways (Bathaie et al., 2012). Despite major advancement in diabetes treatment over the past 3 decades, the treatment outcome of treatment is far from perfect. Drug resistance, debilitating side effects, severe toxicity, and drug contraindications due to *in vitro* interactions are all problems that these treatments face (Kooti et al., 2016). Medicinal plant treatment regimens have been proposed by a number of researchers (Kooti et al., 2015). Hundreds of plants have been documented to have positive anti-diabetic effects, including *Acacia Arabica* (Hegazy et al., 2013), *Allium sativum* (Kazi, 2014)*, Azadirachta indica* (Hashmat et al., 2012), *Blighia sapida* (Ojo et al., 2017), and *Caesalpinia bonducella* (Nazeerullah et al., 2012).

*Cenchrus purpureus* (Schumach.) Morrone (family: Poaceae) is a major multipurpose high yielding tropical grass also known as elephant grass, Napier grass, Uganda grass, among other names. It is a highly adaptable species that can thrive in a variety of environments and agricultural systems, and it is found all over the tropics (FAO, 2015). Because of its high productivity, elephant grass is a very important forage plant in the tropics. It is high in fiber, essential nutrients, and trace minerals, with a protein content that’s typical of forage plants (Evitayani et al., 2004). Soups and stews can be made with the young shoots (Akah and Onweluzo, 2014). Elephant grass, as implied by its name, is an important source of food for elephants in Africa (Francis, 2004) and is suited to feed cattle and buffaloes. *C. purpureus* is a fast-growing and high biomass-producing cellulosic source and has been a choice candidate for biofuel feedstock (Rocha et al., 2017) and weed control where it is used as a trap plant in push-pull management strategies to fight against stem borers in maize crops (Khan et al., 2007). Although there is less research and documentation on its efficacy as a medicinal plant in the treatment of various ailments, there are reports that the shoot and culm infusions have diuretic properties (Duke, 1983). Its phytochemical composition also revealed a higher proportion of terpenoids, alkaloids, calcium (Prinsen et al., 2012), and riboflavin than many vegetables including onion, cabbage, carrot, cauliflower, cucumber, chili pepper, and spinach (Burkill, 1985). *C. purpureus* was reported by Okaraonye and Ikewuchi, 2009 to contain alkaloids, cyanogenic glycosides, flavonoids, saponins, and tannins. *C. purpureus* has been reported as a traditional treatment for diabetes mellitus (Akuru et al., 2015). *C. purpureus* has been discovered to be a powerful antioxidant and hypoglycemic agent (Tsai et al., 2008; Okaraonye and Ikewuchi, 2009; Olorunsanya et al., 2011; Akuru et al., 2015; Tjeck et al., 2017). Although previous research has shown that *C. purpureus* shoot extract (CPSE) has anti-diabetic properties, its mechanism has yet to be investigated. As a result, this study used a diabetic rat model to assess the effects of AECPS on pancreatic-cell dysfunction and apoptosis, as well as to investigate the mechanism of action of *C. purpureus* in alloxan-induced diabetic rats.

## 2 Materials and Methods

### 2.1 Chemicals

Alloxan, α-amylase, diphenylamine, acarbose, and α-glucosidase were products of Sigma-Aldrich (Steinheim, Germany). Enzymes assay kits were products of Randox Laboratories Ltd., Antrim, United Kingdom. Methanol and Folin-Ciocalteu reagent were purchased from Merck (Darmstadt, Germany). All chemical agents and standards were of analytical quality unless otherwise specified.

### 2.2 Plant Material

Shoots of *Cenchrus purpureus* (Schumach.) Morrone were obtained from an agricultural establishment in Ado-Ekiti, Ekiti State, and identified at the Forestry Research Institute of Nigeria (FRIN) by Mr. Odewo with FHI 113161 given as the herbarium number.

The http://mpns.kew.org/mpns-portal/?_ga=1.111763972.1427522246.1459077346 link was used to check and confirm the accepted name of the plant.

### 2.3 Preparation of Aqueous Extract of Cenchrus Purpureus Shoots

The shoots were chopped into small pieces and dried to constant weight, for 4 weeks at ambient temperature (approximately 25°C), after which it was ground into powder with an electric blender (Kenwood, Model BL490, China). 100 g of dried shoots was soaked in distilled water for 48 h to obtain an aqueous extract (Ojo et al., 2017). We lyophilized the resulting aqueous extract (Modulyo Freeze Dryer, Edward, England) to yield 15.5 g.

### 2.4 High-Performance Liquid Chromatography Analysis of AECPS

HPLC analysis of AECPS was determined by the chromatographic system (N2000) comprising Autosampler (YL 9150) with a 100 μl fixed loop and a YL9120 UV-visible detector. We did the separation on an SGE Protocol PC18GP120 (250 mm × 4.6 mm, 5 μm) column at 25°C. The mobile phase was methanol to water (70:30 v/v), and we achieved the separation utilizing the isocratic mode. The elution flow rate was 1 ml/min. Samples were run for 15 min, and we achieved detection at 254 and 366 nm. Stock solutions of standards references were prepared in the HPLC mobile phase at a concentration range of 0.030–0.500 mg/ml. Chromatography peaks were confirmed by comparing its retention time with those of reference standards.

### 2.5 *α*-Amylase and *α*-Glucosidase Inhibitory Activities

The protocol described by Shai et al., 2010 and Nguelefack, et al., 2020 was used to estimate the inhibitory activities of AECPS against the α-amylase and α-glucosidase enzymes at different concentrations ranging from 15–240 μg/ml. The standard employed for this study was acarbose. The result was calculated and expressed as a percentage.

### 2.6 Experimental Animals and Dosage Determination

Thirty-six (36) male experimental Wistar rats (252.50 ± 10.52 g) were bought from the Animal Holding Unit of the Department of Biochemistry, University of Ilorin, in Ilorin, Kwara State. Male rats were selected because they have more stable hormonal status in comparism to female rats. Also, male rats tend to develop more pronounced insulin resistance whilst females show a greater loss of insulin release and beta cell mass. The experimental animals were kept in cages at a temperature of 22–30°C, a photoperiod of 12 h natural light and 12 h darkness, and a relative humidity between 40–45%. They were fed animal feed (Top Feeds, Beside First Bank Plc, Adebayo, Ado-Ekiti, Nigeria) and freely available tap water. The animals were acclimatized for 2 weeks before the start of the experiment. An ethnobotanical survey and personal communications with traditional medicine practitioners revealed that about 200 ml of the juice extract should be administered two times a day for effective treatment of diabetes by a patient weighing about 70 kg (Yakubu et al., 2016; Ajiboye et al., 2021). However, 2.92 g of AECPS extract was derived after freeze-drying 200 ml of the juice. And from this extrapolation, 8.4 mg/kg body wt. was adopted as a dosage to test the anti-hyperglycemic potential of the extract.

### 2.7 DM Induction

The protocol previously employed by Ojo et al., 2017, induced DM in experimental rats. Male Wistar rats 36) fasted without food but with water available for 12 h. The fasting blood sugar level of the rats was checked before the injection of alloxan. Then, 30 male Wistar rats were given a 65 mg/kg of body weight single injection (I.P) of alloxan (2 g) dissolved in normal saline (NaCl) and a 5% glucose solution to induce insulin resistance. The fasting blood sugar (FBS) level for each induced animal was checked after 72 h to confirm the induction of DM (Murat and Serfaty, 1974). Animals having an FBS level >250 mg/dl were diabetic.

### 2.8 Animal Groups and Treatment With Extract

Thirty-six male Wistar rats were chosen and divided into six groups of six rats each. The groupings were:

Group 1: Normal rats + distilled water.

Group 2: Diabetic control rats + distilled water.

Group 3: Diabetic rats +30 mg/kg b. wt of metformin.

Group 4: Diabetic rats +4.2 mg/kg b. wt of AECPS.

Group 5: Diabetic rats +8.4 mg/kg b. wt of AECPS.

Group 6: Diabetic rats +16.8 mg/kg b. wt of AECPS.

#### 2.8.1 Ethical Approval

All experimental rats that were used for this study were handled in line with the rules and regulations for animal management used in the research as contained in ARRIVE guidelines prepared for the care and use of animals in a laboratory. In addition, the Landmark University Ethical Committee approved this research and gave this approval number: LUAC/2021/006A.

### 2.9 Collection and Analysis of Samples

This research experiment lasted 2 weeks, after which the rats were euthanized using halothane anesthesia and sacrificed through cervical dislocation; the liver and pancreas were harvested and homogenized in cold phosphate buffer (0.01M, pH 7.4, 1:5 w/v) before being kept at a temperature of −4°C following the protocol by Ojo et al., 2017. Blood was drawn from the jugular veins, placed in a clean dry centrifuge tube, allowed to clot at room temperature before being centrifuged at 5,000 rpm for 15 min, and sera were preserved for further analysis. The tissues were centrifuged for 15 min at 5,000 rpm, and the supernatant were separated with Pasteur pipettes, transferred into specimen bottles and frozen at −80°C for further biochemical analysis.

### 2.10 Biochemical Parameters

Serum insulin concentration determination was achieved based on the method described by Ibrahim and Islam, 2014, which used an ELISA kit from Sweden in a multiple plate ELISA reader (Winooski, Vermont, United States). The other biochemical parameters were determined using the method described for liver glycogen (Murat and Serfaty, 1974), serum total cholesterol, triglyceride, HDL-cholesterol (Fredrickson et al., 1967), LDL and VLDL cholesterol (Friedwald et al., 1972) respectively. The atherogenic index (AI) and coronary artery index (CRI) were calculated by using the expression in Liu et al., 1999 and Boers et al., 2003. The homeostasis model assessment of insulin resistance (HOMA-IR) and homeostasis model assessment of the *ß*-cell score (HOMA-*β*) were determined using the expression (1) and (2) described by Ibrahim and Islam, 2014.

$$\mathrm{HOMA}-\mathrm{IR}=\frac{[insulin\;\left(U/L\right)X\;blood\;glucose\left(mmol/L\right)}{22.5}$$ (1)

$$\mathrm{HOMA}-\beta =\frac{\left[20\;X\;insulin\;\left(U/L\right)\right]}{\left[blood\;glucose\;\left(mmol/L\right)-3.5\right]}$$ (2)

Converting factors for units: insulin (1 U/L = 7.174 pmol/L) and blood glucose (1 mmol/L = 18 mg/dl).

### 2.11 Determination of Biomarkers of Oxidative Stress

The supernatants of both the liver and pancreas were used to assay for reduced glutathione (GSH) level (Livingstone and Davis, 2007), catalase, superoxide dismutase activities (Zelko et al., 2002), and malondialdehyde MDA) level (Varshney and Kale, 1990).

### 2.12 Determination of Liver Glycogen and the Activities of Glycolytic Enzymes

Liver glycogen was determined following the protocol described by Morales et al., 1973. The liver supernatant was used to analyze the activities of glycolytic enzyme activities which include: hexokinase, glucose-6-phosphatase (G6P) (Erukainure et al., 2017), and fructose 1,6-bisphosphatease (F-1,6-BPase) (Gancedo and Gancedo., 1971).

### 2.13 Determination of Inflammatory Biomarkers

TNF-α, IL-6, as well as NF-κB, were determined in the serum using the protocol outlined in ELISA kits (Biosource, United States).

### 2.14 Quantitative RT PCR (RT-PCR) Analysis

#### 2.14.1 Total RNA Isolation

We removed the total RNA from the entire organs following a technique described by Omotuyi et al., 2018.

#### 2.14.2 cDNA Transformation

Before cDNA transformation, the absolute RNA amount (concentration (µg/ml) = 40 * A_*260*_) and quality (≥1.8) were evaluated using the proportion of A_260_/A_280_ (A = absorbance) read via a spectrophotometer (Jen-way UV-VIS spectrophotometer model 6,305, United Kingdom).

#### 2.14.3 PCR Amplification and Agarose Gel Electrophoresis

PCR amplification for the assessment of genes whose primers (Primer3 software) are recorded below was performed using the procedure described by Omotuyi et al., 2018.

PI3K mRNA Sequence (5′->3′)

Forward primer GGT​GCT​AAG​GAG​GAG​CAC​TG.

Reverse primer CCA​TGT​GGT​ACA​GGC​CAG​AG.

AKT mRNA Sequence (5′->3′)

Forward primer AAG​GAC​CCT​ACA​CAG​AGG​CT.

Reverse primer AAG​GTG​GGC​TCA​GCT​TCT​TC.

GAPDH mRNA Sequence (5′->3′)

Forward primer GCA​TCT​TCT​TGT​GCA​GTG​CC.

Reverse primer GAG​AAG​GCA​GCC​CTG​GTA​AC.

Bcl-2 mRNA Sequence (5′->3′)

Forward primer GCG​TCA​ACA​GGG​AGA​TGT​CA.

Reverse primer TTC​CAC​AAA​GGC​ATC​CCA​GC.

PCNA mRNA Sequence (5′->3′)

Forward primer AGC​AAC​TTG​GAA​TCC​CAG​AAC​A.

Reverse primer CAC​AGG​AGA​TCA​CCA​CAG​CA.

Cyclophilin A mRNA Sequence (5′->3′)

Forward primer TGG​AGA​GCA​CCA​AGA​CAG​ACA.

Reverse primer TGC​CGG​AGT​CGA​CAA​TGA​T.

##### 2.14.4 Amplicon Image Processing

Photographs of the in-gel amplicon bands were treated on a Keynote platform, as revealed by Omotuyi, et al., 2018, and evaluated via ImageJ software.

### 2.15 Data Analysis

We conducted the *in vitro* studies in triplicates. For the *in vivo* analysis, the data were all interpreted as mean ± standard deviation (SD) for six readings across the groups. We then subjected the data from this study to analysis using one-way analyses of variance. Tukey’s *post hoc* comparison test was done with GraphPad Prism nine version at a significance level of *p* < 0.05.

## 3 Results

### 3.1 HPLC-UV Analysis of Aqueous Extract of *C. purpureus* Shoots

The HPLC analysis of AECPS (Supplementary Figure S1) revealed the presence of five constituents with different retention times. Table 1 shows that five active compounds, 3-O-rutinoside, ellagic acid, catechin, rutin and kaempferol were identified in AECPS; these may have resulted in some of the therapeutic effects observed from AECPS in this study.

**TABLE 1:** Bioactive compounds identified in AECPS.

Compounds	Retention time	Concentration (µg/10 g)
3-O-rutinoside	1.532	209.9641
Ellagic acid	0.132	3.0359
Catechin	0.095	2.3760
Rutin	0.084	2.1022
Kaempferol	0.031	0.9120

Legends: AECPS: aqueous extract of *cenchrus purpureus* shoots.

### 3.2 α-Glucosidase and α-Amylase Inhibitory Activities of AECPS

**FIGURE 1:**
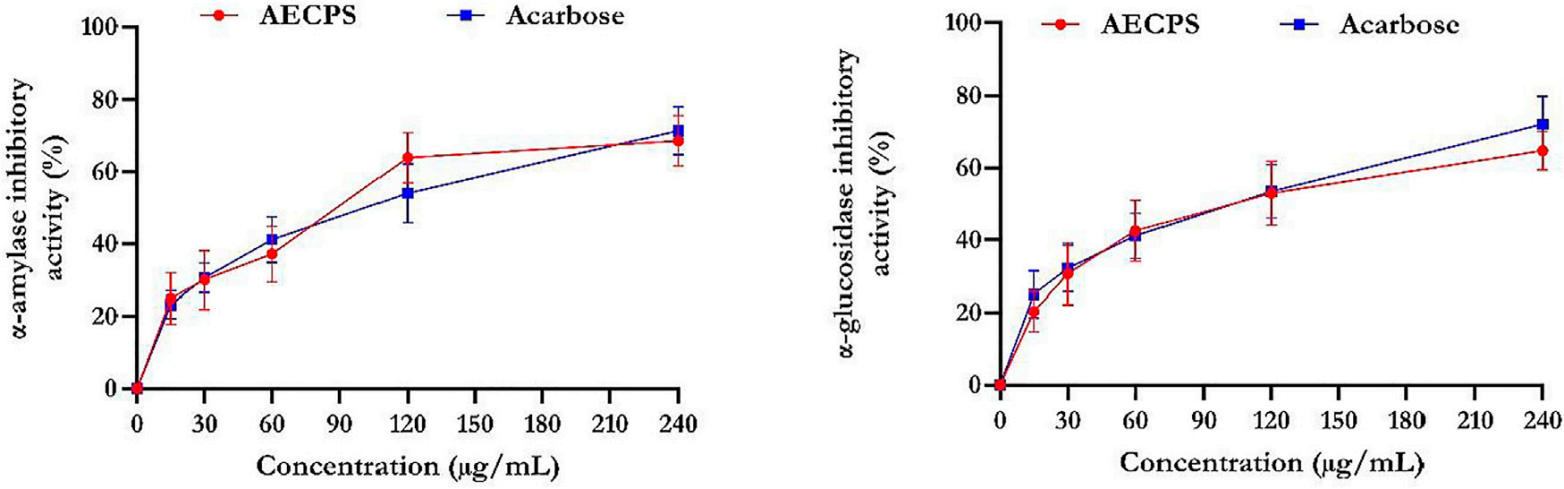
α-glucosidase and α-amylase inhibitory activities of aqueous extract of C. purpureus shoots Data are expressed as mean ± SD of triplicates determinations Legend: AECPS: aqueous extract of *Cenchrus purpureus* shoots.

Figure 1 shows that acarbose had a superior inhibitory activity for α-glucosidase compared with that of AECPS. AECPS exhibited its maximal αglucosidase inhibitory activity at 60% (IC_50_ = 3.70 μg/ml) and acarbose at 68% (IC_50_ = 3.45 μg/ml). In addition, the α**-**amylase inhibitory property exhibited by AECPS was 63% compared to 65% for acarbose with IC_50_ values of 3.42 μg/ml and 3.50 μg/ml respectively. This suggest the antidiabetic activity of AECPS.

### 3.3 Fasting Blood Glucose Level of Diabetic Rats Administered AECPS

Before we administered alloxan, all the animals in all the experimental groups had normal blood glucose levels (Table 2). Blood glucose levels in all groups increased 48 h after alloxan administration, exceeding those in the normal control group that did not receive alloxan. After days 7 and 14, all treatment groups had lower blood glucose levels when compared to the diabetic control group. Administration of AECPS at 4.2,8.4 and 16.8 mg/kg body weight significantly (*p* < 0.05) and progressively reduced the blood glucose levels by 63%, 69% and 77% respectively as against 80% by metformin. After 14 days, the reduction in blood sugar levels in the AECPS (16.8 mg/kg)-treated group were comparable to those in the metformin-treated group. The diabetic control group’s blood glucose levels continued to rise over the course of the 14 days experiment, whereas the normal control group’s blood glucose levels remained within normal limits.

**TABLE 2:** Fasting blood glucose (mg/dl) levels of alloxan-induced diabetic rats before and after oral administration of aqueous extract of C. purpureus shoots.

Treatment groups	Initial FBG value (mg/dl)	FBG value after 48 h of induction (mg/dl)	FBG value after 7 days of treatment (mg/dl)	FBG value after 14 days of treatment (mg/dl)
Normal Control	69.91 ± 3.86^a^	69.91 ± 3.86^a^	69.91 ± 3.86^a^	79.92 ± 5.33^a^
Diabetic Control	66.16 ± 6.44^c^	365.38 ± 94.12^b^	393.66 ± 86.24^d^	436.14 ± 37.83^b^
Diabetic + Metformin	63.25 ± 2.13^b^	408.15 ± 98.51^c^	225.13 ± 61.22^c^	87.30 ± 9.93^c^
Diabetic + AECPS (4.2 mg/kg)	65.09 ± 2.61^d^	441.59 ± 101.07^c^	200.96 ± 26.35^c^	160.30 ± 26.10^d^
Diabetic + AECPS (8.4 mg/kg)	60.84 ± 2.44^b^ ^,^ ^c^	495.30 ± 118.55^c^	216.20 ± 59.44^c^	134.41 ± 22.56^d^
Diabetic + AECPS (16.8 mg/kg)	65.93 ± 2.43^c^	496.61 ± 133.32^c^	194.31 ± 52.67^b^	98.32 ± 19.91^c^

Data are expressed as mean ± SD (*n* = 6). Down the column.

a values with different letters are significantly different (*p* < 0.05) from each other.

b values with different letters are significantly different (*p* < 0.05) from each other.

c values with different letters are significantly different (*p* < 0.05) from each other.

d values with different letters are significantly different (*p* < 0.05) from each other.

*AECPS: Aqueous extract of *C. purpureus* shoots; FBG: fasting blood glucose.

### 3.4 Body Weight of Diabetic Rats Administered AECPS

The results in Table 3 show the bodyweight of animals before and after oral administration of AECPS. Over 8% weight loss was observed in the diabetic control group, while over 10% weight loss was also observed in the metformin-treated group. The normal control group showed a significant increase (6.85%) in animal body weight after 14 days as did the 16.8 mg/kg AECPS treated group, which showed a percentage weight gain of 4.84%. We observed no significant difference in the organ-body weight for the liver and pancreas of any of the control and treated groups, as shown in Table 4.

**TABLE 3:** Body weight of alloxan-induced diabetic rats before and after oral administration of aqueous extract of C. purpureus shoots.

Treatment groups	Initial weight (g)	Final weight (g)	% weight change
Normal Control	243.26 ± 24.87	259.45 ± 15.83^c^	6.85↑
Diabetic Control	242.06 ± 24.08	222.33 ± 20.29^b^ ^,^ ^c^	8.15↓
Diabetic + Metformin	245.33 ± 21.07	216.58 ± 26.47^b^ ^,^ ^c^	11.72↓
Diabetic + AECPS (4.2 mg/kg)	248.15 ± 20.69	234.23 ± 21.44^a^	7.92↓
Diabetic + AECPS (8.4 mg/kg)	248.10 ± 23.95	271.11 ± 26.27^a^ ^,^ ^b^	15.25↑
Diabetic + AECPS (16.8 mg/kg)	249.11 ± 24.99	256.50 ± 27.22^a^	4.84↑

Data are expressed as mean ± SD *(n* = 6).

a Values with different letters along a column for a given parameter are significantly different (*p* < 0.05) from each other.

b Values with different letters along a column for a given parameter are significantly different (*p* < 0.05) from each other.

c Values with different letters along a column for a given parameter are significantly different (*p* < 0.05) from each other.

AECPS: aqueous extract of *cenchrus purpureus* shoots; * Weight loss (↓); *Weight gain (↑).

**TABLE 4:** Body weight, organ weight and organ-body weight ratios of alloxan-induced male Wistar rats administered orally with aqueous extract of C. purpureus shoots.

Groups	Parameters					
	Initial body weight (g)	Final body weight (g)	Weight of liver (g)	Weight of pancreas (g)	LIVER-BODY weight (%)	PANCREAS-BODY weight (%)
Normal Control	243.26 ± 24.87	259.45 ± 15.83^c^	6.26 ± 1.04^b^	0.45 ± 0.06^c^	2.48 ± 0.14^a^	0.18 ± 0.05^a^
Diabetic Control	242.06 ± 24.08	222.33 ± 20.29^b^ ^,^ ^c^	7.04 ± 1.67^b^	0.37 ± 0.05^b^ ^,c^	3.58 ± 1.08^a^	0.20 ± 0.07^a^
Diabetic + Metformin	245.33 ± 21.07	216.58 ± 26.47^b^ ^,^ ^c^	6.67 ± 1.15^b^	0.24 ± 0.25^a^ ^,b^	2.91 ± 0.09^a^	0.11 ± 0.02^a^
Diabetic + AECPS (4.2 mg/kg)	248.15 ± 20.69	234.23 ± 21.44^a^	3.78 ± 0.47^a^	0.19 ± 0.01^a^	4.43 ± 0.67^a^	0.19 ± 0.03^a^
Diabetic + AECPS (8.4 mg/kg)	248.10 ± 23.95	271.11 ± 26.27^a^ ^,^ ^b^	3.78 ± 0.49^a^	0.16 ± 0.02^a^	3.31 ± 0.37^a^	0.12 ± 0.02^a^
Diabetic + AECPS (16.8 mg/kg)	249.11 ± 24.99	256.50 ± 27.22^a^	7.08 ± 1.35^b^	0.19 ± 0.17^a^	4.30 ± 0.72^a^	0.14 ± 0.05^a^

Data are expressed as mean ± SD (*n* = 6).

a Values with different letters along a column for a given parameter are significantly different (*p* < 0.05) from each other.

b Values with different letters along a column for a given parameter are significantly different (*p* < 0.05) from each other.

c Values with different letters along a column for a given parameter are significantly different (*p* < 0.05) from each other.

*AECPS: Aqueous extract of *C. purpureus* shoots.

### 3.5 Serum Insulin Levels, HOMA-IR and HOMA- *ß* Levels of Diabetic Rats Administered AECPS

Table 5 shows the serum insulin, HOMA-IR, and HOMA-β scores in the diabetic rats. Induction of diabetes significantly reduced the levels of serum insulin, whereas metformin and AECPS administration increased it, with AECPS (16.8 mg/kg) comparing well with the normal controls. AECPS increased serum insulin in a dose-dependent manner. HOMA-IR increased after the induction of diabetes but treatment with metformin and AECPS reduced it as all the treatment groups to a level which compared well with the control group. In contrast, the HOMA-β score was reduced in diabetic rats whereas AECPS and metformin increased the scores in the treated groups, which all compared favourably to the normal control group.

**TABLE 5:** Serum insulin levels, HOMA-IR and HOMA-*β* scores of alloxan-induced diabetic rats after oral administration of aqueous extract of C. purpureus shoots.

Groups	Parameters		
	Insulin (U/l)	HOMA-IR	HOMA-*β*
Normal Control	10.43 ± 0.07^e^	2.01 ± 0.13^a^ ^,^ ^b^	44.74 ± 3.74^b^ ^,^ ^c^
Diabetic Control	5.11 ± 0.69^a^	6.34 ± 0.65^c^	0.17 ± 0.02^a^
Diabetic + Metformin	8.25 ± 1.00^b^	1.71 ± 0.80^a^	31.87 ± 1.85^b^
Diabetic + AECPS (4.2 mg/kg)	9.07 ± 1.05^c^	1.37 ± 0.61^a^	53.16 ± 1.96^c^
Diabetic + AECPS (8.4 mg/kg)	9.67 ± 0.68^c^ ^,^ ^d^	2.27 ± 0.81^a^ ^,^ ^b^	33.34 ± 1.33^b^ ^,^ ^c^
Diabetic + AECPS (16.8 mg/kg)	10.29 ± 1.14^d^ ^,^ ^e^	3.14 ± 0.42^b^	26.84 ± 2.54^b^

Data are expressed as mean ± SD (*n* = 6).

a Values with different letters along a column for a given parameter are significantly different (*p* < 0.05) from each other.

b Values with different letters along a column for a given parameter are significantly different (*p* < 0.05) from each other.

c Values with different letters along a column for a given parameter are significantly different (*p* < 0.05) from each other.

d Values with different letters along a column for a given parameter are significantly different (*p* < 0.05) from each other.

e Values with different letters along a column for a given parameter are significantly different (*p* < 0.05) from each other.

*AECPS: Aqueous extract of C. purpureus shoots.

*HOMA-IR (Homeostatic model assessment of insulin resistance): [(Fasting serum insulin in U/l *fasting blood glucose in mmol/l)/22.5].

*HOMA-β (Homeostatic model assessment of β-cell function: [(Fasting serum insulin in U/l *20/fasting blood glucose in mmol/l-3.5)].

*Conversion factor: Insulin (1U/l = 7.174 pmol/l).

### 3.6 Antioxidant Markers in Experimental DM

When compared to the normal control group, alloxan significantly increased hepatic MDA levels in diabetic control rats, but all treatment groups (including metformin) significantly reduced hepatic MDA to a level comparable to the normal control group (Table 6). MDA levels in the AECPS-treated groups decreased in a dose-dependent manner. In contrast, alloxan induction significantly decreased the activities of the CAT, SOD, GPX, and GST enzymes as well as the levels of GSH, however, metformin increased these antioxidant parameters in the metformin-treated group but not to levels comparable with the normal controls. The antioxidant parameters were also increased in the AECPS-treated group in a dose-dependent manner, with AECPS (16.8 mg/kg) providing the best result, which also compared favorably to the normal control.

**TABLE 6:** Hepatic antioxidant markers of alloxan-induced diabetic rats after oral administration of aqueous extract of C. purpureus shoots.

Groups	Parameters					
	MDA (nmol/mg protein)	CAT (U/mg protein)	SOD (U/mg protein)	GPX (U/mg protein)	GSH (µmol/mg tissue)	GST (U/mg protein)
Normal Control	0.05 ± 0.01^a^	10.28 ± 0.02^f^	4.90 ± 0.63^c^	7.98 ± 0.11^b^	10.27 ± 1.43^e^	11.20 ± 1.81^b^
Diabetic Control	2.66 ± 0.46^b^	1.14 ± 0.01^a^	1.12 ± 0.01^a^	1.56 ± 0.23^a^	2.18 ± 0.01^a^	1.10 ± 0.03^a^
Diabetic + Metformin	0.07 ± 0.01^a^	7.09 ± 0.02^d^	4.86 ± 0.02^c^	6.21 ± 0.01^c^	8.27 ± 0.02^d^	9.32 ± 0.04^c^
Diabetic + AECPS (4.2 mg/kg)	0.19 ± 0.03^a^	5.61 ± 0.43^b^	2.37 ± 0.01^b^	3.09 ± 0.03^d^	5.20 ± 0.01^b^	6.75 ± 0.06^d^
Diabetic + AECPS (8.4 mg/kg)	0.15 ± 0.02^a^	6.89 ± 0.44^e^	5.09 ± 0.01^e^	5.72 ± 1.46^e^	7.22 ± 0.02^c^	7.58 ± 1.27^e^
Diabetic + AECPS (16.8 mg/kg)	0.11 ± 0.01^a^	9.14 ± 0.18^e^	7.15 ± 0.02^d^	6.19 ± 1.72^c^	10.21 ± 1.22^e^	11.19 ± 1.34^b^ ^,^ ^f^

Data are expressed as mean ± SD (*n* = 6).

a Values with different letters along a column for a given parameter are significantly different (*p* < 0.05) from each other.

b Values with different letters along a column for a given parameter are significantly different (*p* < 0.05) from each other.

c Values with different letters along a column for a given parameter are significantly different (*p* < 0.05) from each other.

d Values with different letters along a column for a given parameter are significantly different (*p* < 0.05) from each other.

e Values with different letters along a column for a given parameter are significantly different (*p* < 0.05) from each other.

f Values with different letters along a column for a given parameter are significantly different (*p* < 0.05) from each other.

*AECPS: Aqueous extract of *C. purpureus* shoots.

*MDA: malondialdehyde, *CAT: catalase, *SOD: superoxide dismutase, *GPX: glutathione peroxidase.

* GSH: Reduced Glutathione. *GST: Glutathione s-transferase.

Alloxan also significantly increased pancreatic MDA levels in the diabetic control rats, but all treatments significantly reduced it, with metformin and AECPS at 8.4 mg/kg and 16.8 mg/kg, respectively, comparing favorably with the normal control group (Table 7). The reduction in the extract-treated group was in a dose-dependent manner. Alloxan also significantly reduced the pancreatic antioxidant enzyme activities and GSH levels in the diabetic control group, but the treatment groups (including metformin) increased it. The group treated with AECPS (16.8 mg/kg) and metformin compared well with the normal control.

**TABLE 7:** Pancreatic antioxidant markers of alloxan-induced diabetic rats after oral administration of aqueous extract of C. purpureus shoots.

Groups	Parameters					
	MDA (nmol/mg protein)	CAT (U/mg protein)	SOD (U/mg protein)	GPX (U/mg protein)	GSH (µmol/mg tissue)	GST (U/mg protein)
Normal Control	1.56 ± 0.25^a^	10.11 ± 1.26^f^	5.44 ± 1.01^a^	9.18 ± 0.03^a^	12.12 ± 1.18^e^	11.04 ± 0.90^e^
Diabetic Control	4.84 ± 0.21^c^	2.18 ± 0.01^b^	0.04 ± 0.01^d^	2.19 ± 0.01^d^	4.18 ± 0.44^a^	3.26 ± 0.03^a^
Diabetic + Metformin	1.09 ± 0.01^a^	9.82 ± 0.02^e^	5.11 ± 0.23^a^	8.45 ± 0.02^a^	11.17 ± 0.49^d^	10.14 ± 0.51^d^
Diabetic + AECPS (4.2 mg/kg)	3.05 ± 0.25^b^	5.07 ± 0.11^c^	3.90 ± 0.10^b^	5.23 ± 0.02^c^	8.19 ± 0.56^b^	6.23 ± 0.33^b^
Diabetic + AECPS (8.4 mg/kg)	2.04 ± 0.12^a^ ^,^ ^b^	7.14 ± 0.21^d^	4.44 ± 0.05^e^	7.38 ± 0.01^b^	9.19 ± 0.43^b^ ^,^ ^c^	8.14 ± 0.36^e^
Diabetic + AECPS (16.8 mg/kg)	1.03 ± 0.41^a^	8.30 ± 0.19^d^ ^,^ ^e^	5.14 ± 0.01^a^	8.98 ± 0.01^a^	10.29 ± 0.90^e^	10.32 ± 0.67^d^

Data are expressed as mean ± SD (*n* = 6).

a Values with different letters along a column for a given parameter are significantly different (*p* < 0.05) from each other.

b Values with different letters along a column for a given parameter are significantly different (*p* < 0.05) from each other.

c Values with different letters along a column for a given parameter are significantly different (*p* < 0.05) from each other.

d Values with different letters along a column for a given parameter are significantly different (*p* < 0.05) from each other.

e Values with different letters along a column for a given parameter are significantly different (*p* < 0.05) from each other.

f Values with different letters along a column for a given parameter are significantly different (*p* < 0.05) from each other.

*AECPS: Aqueous extract of *C. purpureus* shoots.

*CAT: catalase; *SOD: superoxide dismutase; *GPX: glutathione peroxidase; *GSH: reduced glutathione.

*GST: Glutathione s-transferase; *MDA: malondialdehyde.

### 3.7 Serum Lipid Parameters in Experimental DM Administered AECPS

As seen in Table 8, total cholesterol was significantly increased in the diabetic control group as compared with the normal control. The different treatment groups reduced total cholesterol with the AECPS treated groups comparing well with those of the normal control. Alloxan significantly decreased HDL-c levels in the diabetic control group, but the different treatment groups increased it. The increase in groups treated with AECPS was in a dose-dependent manner with the AECPS (16.8 mg/kg) treated group comparing well to the normal control. Alloxan administration increased TG, VLDL-c, LDL-c, AI, and CRI, separately, but the administration of metformin and AECPS reduced them. The AECPS treated groups (16.8 mg/kg and 8.4 mg/kg) compared well to the normal controls. All the treatment groups also compared well to the normal control in reducing AI and CRI levels.

**TABLE 8:** Lipid profile of alloxan-induced diabetic rats after oral administration of aqueous extract of C. purpureus shoots.

Groups	Parameters						
	TC (mmol/l)	HDL-c (mmol/l)	TG (mmol/l)	VLDL-c (mmol/l)	LDL-c (mmol/l)	AI	CRI
Normal Control	45.90 ± 4.38^a^	33.94 ± 5.66^d^	28.42 ± 2.23^a^	5.68 ± 0.44^a^	6.27 ± 3.14^a^	0.35 ± 0.08^a^	1.35 ± 0.13^a^
Diabetic Control	104.25 ± 7.50^c^	5.88 ± 0.47^a^	72.37 ± 0.47^e^	14.47 ± 0.05^e^	83.90 ± 6.98^d^	17.38 ± 1.00^b^	18.38 ± 0.40^b^
Diabetic + Metformin	71.12 ± 8.78^b^	20.23 ± 0.67^b^	61.28 ± 0.52^d^	12.26 ± 0.10^d^	38.63 ± 9.10^c^	2.52 ± 0.05^a^	3.52 ± 0.47^a^
Diabetic + AECPS (4.2 mg/kg)	57.18 ± 4.57^a^ ^,^ ^b^	23.94 ± 0.31^b^ ^,^ ^c^	51.06 ± 0.22^c^	10.21 ± 0.04^c^	23.02 ± 0.82^b^	1.40 ± 0.01^a^	2.40 ± 0.05^a^
Diabetic + AECPS (8.4 mg/kg)	48.81 ± 1.82^a^	25.29 ± 0.05^b^ ^,^ ^c^	46.00 ± 0.07^c^	9.20 ± 0.01^c^	14.32 ± 1.63^a^ ^,^ ^b^	0.94 ± 0.02^a^	1.94 ± 0.06^a^
Diabetic + AECPS (16.8 mg/kg)	41.91 ± 1.67^a^	28.97 ± 0.87^c^ ^,^ ^d^	39.40 ± 1.73^b^	7.88 ± 0.15^b^	5.06 ± 1.44^a^	0.45 ± 0.03^a^	1.45 ± 0.04^a^

Data are expressed as mean ± SD (*n* = 6).

a Values with different letters along a column for a given parameter are significantly different (*p* < 0.05) from each other.

b Values with different letters along a column for a given parameter are significantly different (*p* < 0.05) from each other.

c Values with different letters along a column for a given parameter are significantly different (*p* < 0.05) from each other.

d Values with different letters along a column for a given parameter are significantly different (*p* < 0.05) from each other.

*AECPS: Aqueous extract of *C. purpureus* shoots.

*TC (Total cholesterol); TG (Triglyceride); *HDL-c (High density lipoprotein-cholesterol).

*AI (Atherogenic index): [(TC-HDL-c)/HDL-c].

*CRI (Coronary index): [(TC (mg/dl)/HDL-c (mg/dl)].

*VLDL-c (Very low density lipoprotein-cholesterol): [TG/5].

*LDL-c (Low density lipoprotein-cholesterol): [TC-HDL-(TG/5)].

### 3.8 Hepatic Glycogen and Carbohydrate Metabolizing Enzymes in Diabetic Rats Administered AECPS

The level of hepatic glycogen and the glycolytic enzyme, hexokinase, were significantly reduced in the diabetic rats, but we observed a notable increase in the treatment groups after administration of metformin and AECPS (Table 9). The activities of the gluconeogenesis enzymes G6P and F-1,6-BP were observed to increase in the diabetic control group, but administration of AECPS and metformin yielded a notable decrease in the treatment groups (Table 9).

**TABLE 9:** Hepatic glycogen and carbohydrate metabolizing enzyme levels after oral administration of aqueous extract of C. purpureus shoots.

Treatment groups	Hepatic GLYCOGEN^α^	HEXOKINASE^β^	FRUCTOSE-1,6-BISPHOSPHATASE^γ^	GLUCOSE-6-PHOSPHATASE^γ^
Normal Control	30.71 ± 1.99^b^	5.92 ± 0.87^e^	3.44 ± 0.06^a^	41.02 ± 2.46^a^
Diabetic Control	8.34 ± 2.97^a^	0.96 ± 0.07^a^	9.85 ± 0.56^c^	77.81 ± 4.28^c^
Diabetic + Metformin	56.03 ± 3.74^d^	4.29 ± 0.12^d^ ^,^ ^e^	3.78 ± 0.05^a^	47.04 ± 4.01^a^
Diabetic + AECPS (4.2 mg/kg)	40.68 ± 1.49^c^	2.98 ± 0.23^b^	2.29 ± 0.12^b^	63.18 ± 3.23^d^
Diabetic + AECPS (8.4 mg/kg)	52.89 ± 0.99^d^	3.11 ± 0.12^c^	2.89 ± 0.19^b^	57.24 ± 2.27^c^
Diabetic + AECPS (16.8 mg/kg)	55.64 ± 4.80^d^	3.97 ± 0.12^c^	3.56 ± 0.18^a^	40.88 ± 5.51^a^

Data are expressed as mean ± SD (*n* = 6).

a Values with different letters along a column for a given parameter are significantly different (*p* < 0.05) from each other.

b Values with different letters along a column for a given parameter are significantly different (*p* < 0.05) from each other.

c Values with different letters along a column for a given parameter are significantly different (*p* < 0.05) from each other.

d Values with different letters along a column for a given parameter are significantly different (*p* < 0.05) from each other.

e Values with different letters along a column for a given parameter are significantly different (*p* < 0.05) from each other.

*AECPS: Aqueous extract of *C. purpureus* shoots.

*^α^: Unit for glycogen (mg of glucose/g of wet tissue).

*^β^: Unit for hexokinase (µmole glucose-6-phosphate formed/min/mg protein).

*^γ^: Unit for fructose-1, 6-bisphosphatase and glucose-6-phosphatase (µmole phosphate liberated/min/mg protein).

### 3.9 Pro- and Anti-Inflammatory Markers of Diabetic Rats Administered AECPS

**FIGURE 2:**
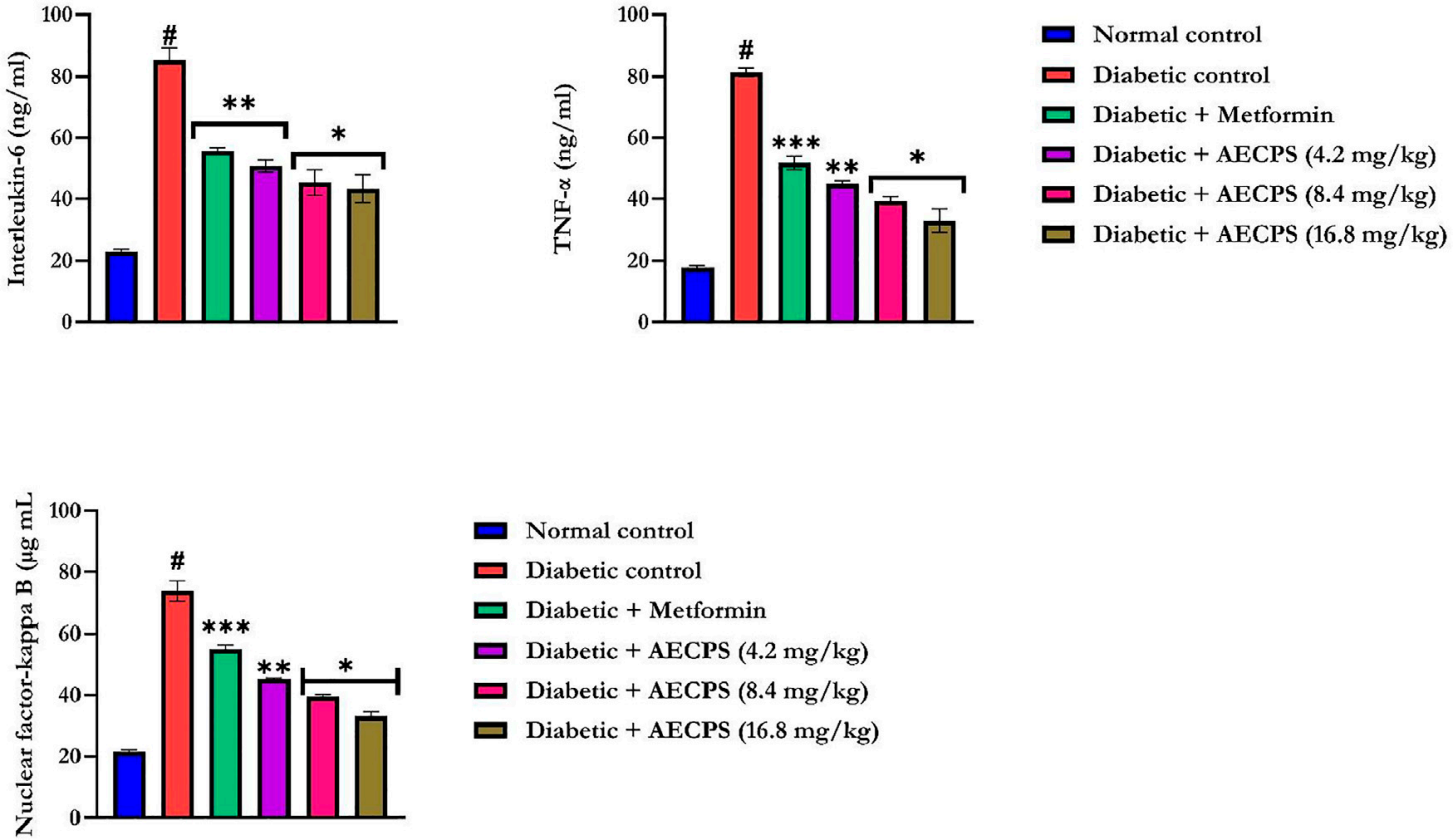
Interleukin-6, Tumor necrosis factor-*α*, and Nuclear factor-kappa B of alloxan-induced diabetic rats orally administered AECPS. Legend: Data are expressed as mean ± SD (*n* = 6); AECPS: aqueous extract of *Cenchrus purpureus* shoots; IL-6: Interleukin-6; TNF-α: Tumor necrosis factor-alpha; NF-κB: Nuclear factor-kappa B; **#**: significantly different from normal control (*p* < 0.05); * is significant at *p* < 0.05 and ** is significant at *p* < 0.01, *** is significant at *p* < 0.001 versus diabetic control.

Administration of alloxan significantly increased the levels of IL-6, TNF- α, and NF-κB (Figure 2) in the rat serum (*p* < 0.05) compared with those of the control rats. AECPS significantly reduced the levels of IL-6, TNF-α, and NF-κB (*p* < 0.05) compared with those in the diabetic rats to levels that were similar to rats treated with metformin. AECPS with 16.8 mg/kg body weight showed the clearest reversal of the alloxan treatment-related rises in the concentrations of IL6, TNF-α, and NF-κB (Figure 2).

### 3.10 Gene Expressions of PI3K, AKT and Apoptotic Markers of Diabetic Rats Administered AECPS

**FIGURE 3:**
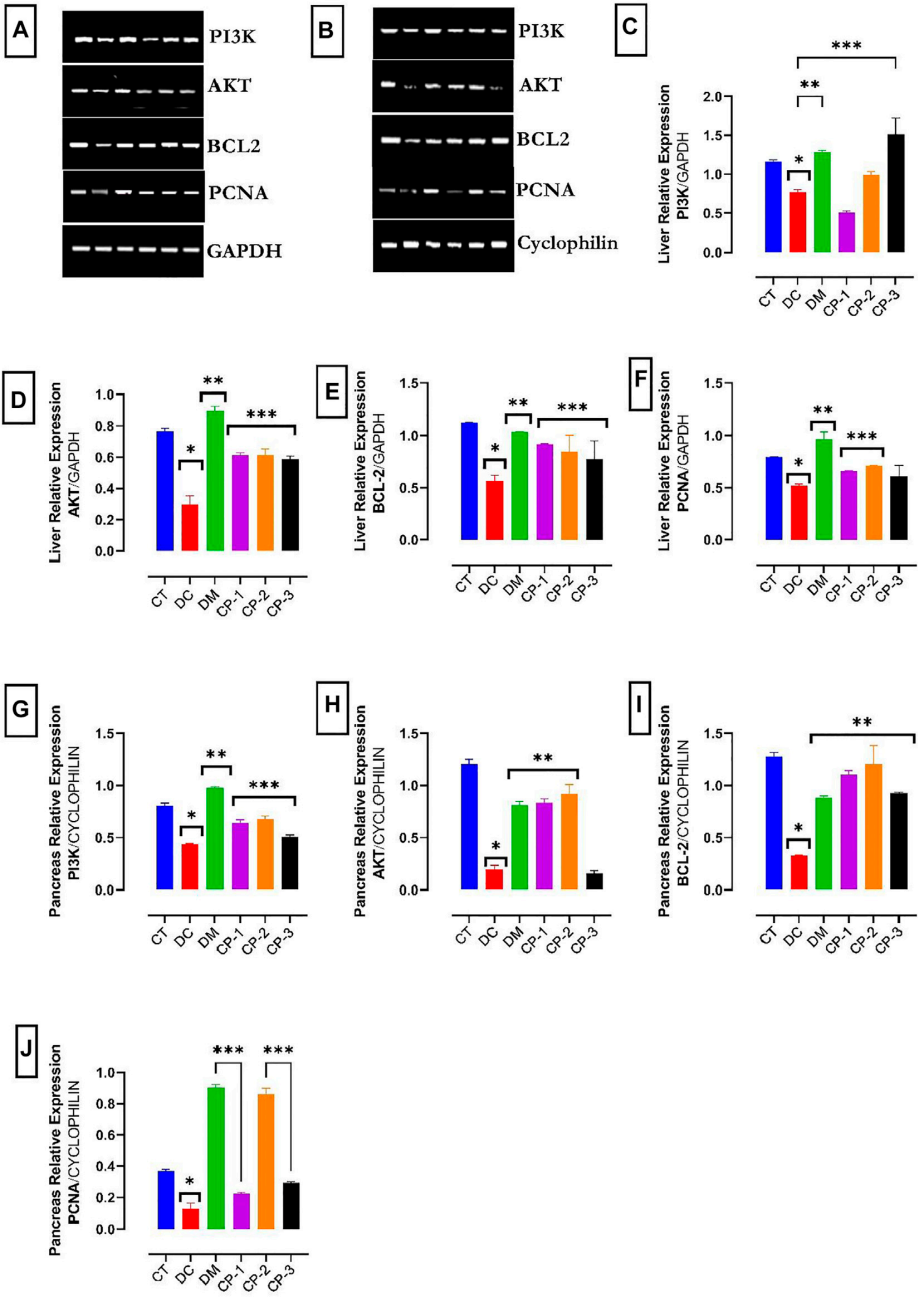
Effect of AECPS on PI3K, AKT, Bcl2 and PCNA in liver and pancreas of diabetic rats in different groups. Data were presented as the mean ± SEM, *n* = 6 for each group **(A)**. mRNA expression of PI3K, AKT, BCL2, and PCNA in liver cells. GAPDH served as control **(B)**. mRNA expression of PI3K, AKT, BCL2, and PCNA in pancreatic cells. Cyclophilin served as control **(C)**. Liver PI3K mRNA expression, *: significantly different from normal control (*p* < 0.05); *p < 0.05 versus control; **p < 0.01 versus DC; ***p < 0.001 versus DC. **(D)** Liver AKT mRNA expression *p < 0.05 versus control; **p < 0.01 versus DC; ***p < 0.001 versus DC. **(E)** Liver Bcl2 mRNA expression, *p < 0.05 versus control; **p < 0.01 versus DC; ***p < 0.001 versus DC. **(F)** Liver PCNA, *p < 0.05 versus control; **p < 0.01 versus Dc; ***p < 0.001 versus DC. **(G)** Pancreas PI3K mRNA expression *p < 0.05 versus control; **p < 0.01 versus DC; ***p < 0.001 versus DC. **(H)** Pancreas AKT mRNA expression, *p < 0.05 versus control; **p < 0.01 versus DC. **(I)** Pancreas Bcl2 mRNA expression, *p < 0.05 versus control; **p < 0.01 versus DC; **(J)** Pancreas PCNA. Legends: AECPS: aqueous extract of *C. purpureus* shoots; PCNA: proliferating cell nuclear antigen; CT: control group; DC: diabetic control; DM: diabetic + metformin group; CP-1: *C. purpureus* treated group (4.2 mg/kg); CP-2: *C. purpureus* treated group (8.4 mg/kg) CP-3: *C. purpureus* treated group (16.8 mg/kg).

The PI3K, AKT, Bcl-2, and PCNA mRNA levels in the liver, and pancreas are shown in Figure 3. The mRNA expression levels of PI3K in the liver and the pancreas were down-regulated in the diabetic rats (Figure 3). In addition, the mRNA levels of AKT, Bcl-2, and PCNA were downregulated in the liver and pancreas of the diabetic rats. Treatment with AECPS and metformin raised the mRNA levels of these compounds in the liver and pancreas.

## 4 Discussion

The phytochemicals in AECPS were identified through HPLC analysis. AECPS contained the flavonoids 3-O-rutinoside, ellagic acid, catechin, rutin and kaempferol were present in AECPS. 3-O-rutinoside has been shown to be an effective inhibitor of α-glucosidase enzyme (Habtemariam, 2011). Ellagic acid works as an anti-diabetic agent by stimulating insulin secretion and decreasing glucose intolerance in pancreatic *ß*-cells (Fatima et al., 2015). Catechin works as an antidiabetic agent by boosting the antioxidant defenses (Samarghandian et al., 2017). Rutin exhibited significant antidiabetic activity, possibly by inhibiting inflammatory cytokines, enhancing antioxidant capacity in a diabetic model and may be useful as a diabetic modulator (Niture et al., 2014), whereas kaempferol may enhance glucose metabolism and inhibit gluconeogenesis (Alkhalidy et al., 2018). As a result, the therapeutic activities of AECPS, as demonstrated by our findings, could be attributed to these compounds identified.

Plants produce phenolic compounds through a series of activities that involve several biosynthetic pathways, including glycolysis, hexose monophosphate shunt, and shikimate pathways in the cytosol (Paucar-Menacho et al., 2017; Ajiboye et al., 2018). They play a significant role in health by regulating weight, metabolism, chronic disease, and cell proliferation (Rodríguez-García et al., 2019). In addition, they possess anti-inflammatory and antioxidant properties that may have preventive and/or curative effects for diseases and disorders, like diabetes mellitus (Adelek et al., 2018a; Rodríguez-García et al., 2019).

Diabetes is an enfeebling chronic metabolic disease with global relevance (Ojo et al., 2017). Diabetes treatment varies depending on the underlying cause of the disease. Insulin can be used to treat type I diabetes, whereas medications that inhibit specific enzymes, such as α-amylase, α-glucosidase, dipeptidyl peptidase IV, and protein tyrosine phosphatase, can be used to treat type II diabetes (Tundis et al., 2010). The primary aim of diabetic treatment is to maintain glycemic control in diabetic patients during both fasting and post-prandial states. Complications from side effects associated with the use of conventional drugs to treat diabetes have prompted a search for alternative anti-diabetic drugs derived from plant (Ojo et al., 2018b). It has been studied to use natural products to either inhibit the production of glucose from carbohydrates in the intestine or the absorption of glucose from the intestine (Ojo et al., 2018b). The α-amylase enzyme can be found in high concentrations in the pancreatic juice and saliva. Salivary *a*-amylase hydrolyzes the α-(1,4)-glycosidic bonds of large insoluble carbohydrates, the primary sources of glucose, into smaller molecules. In contrast, α-glucosidase is localized in the walls of the small intestine and acts by mediating the catabolism of starch and disaccharides into simpler sugars, such as glucose, which are absorbed in the intestine. Alpha-glucosidase regulates postprandial hyperglycemia and is considered to be a potential therapeutic target for diabetes treatment. Inhibiting the activities of these enzymes (*α*-amylase and α-glucosidase) in the gastrointestinal tract (GIT) of humans is known to be an effective diabetic control by lowering postprandial blood glucose (Tundis et al., 2010). The strong inhibitory activity of AECPS against *a*-amylase and *a*-glucosidase found in this study backs up previous reports from other plants (Ojo et al., 2018b). Because the AECPS inhibited the activity of α-glucosidase and α-amylase in a concentration-dependent manner, this inhibition could serve as an indicator of the potential antidiabetic role of the plant.

To reduce the global burden of diabetes mellitus (DM), researchers are searching for newer therapies, especially the use of natural plants, which are readily available and pose fewer or no side effects (Balogun and Ashafa 2017). Some drugs used in treating diabetes are known to have limited efficacy over time (Ojo O. A. et al., 2020). Modern drugs have been reported to losing efficacy over time and attention towards plant materials as s solution to this problem has be of utmost concern. Metformin was used as a standard drug because it has been the preferred and most significant drug for diabetes treatment for over a decade (Balogun and Ashafa 2017). In the current study, we investigated the hypoglycemic potential of AECPS *in vivo* to discover its antidiabetic potency via blood glucose levels.

A significant hallmark of a diabetic state is a reduction in body weight, which can sometimes be ascribed to abnormalities in the catabolism of macronutrients such as fat and protein leading to extreme tissue protein loss and muscle wastage (Balogun and Ashafa 2017). This is clear from our rat diabetic control group, which had a weight loss of more than 8%. AECPS treatment reduced the weight loss even more than the group treated with the standard drug (metformin). The improvement in body weight in the diabetic rats treated with AECPS suggests restoration of tissue and muscle protein as a result of the extract, substantiating the report by Balogun and Ashafa, 2017. The rats treated with AECPS (16.8 mg/kg) showed the highest percentage weight gain, which was higher than that of the normal control. The results from this study did not show any significant change in the organ-body weight ratio (for liver and pancreas) caused by DM even though there was a reduction in overall body weight caused by DM.

DM is a common result of a defect in the secretion or action of insulin and sometimes both Ojo et al., 2017; Ojo O. A. et al., 2020). The administration of the extract not only reduced the blood glucose concentration but also elevated the levels of insulin in the serum. The stimulation of insulin secretion by the extract apparently achieved the reduction in blood glucose level, as other researchers have reported that several plants produce their hypoglycaemic effect in this manner (Balogun and Ashafa 2017). The use of alloxan at a low dose causes partial destruction of the hepatic beta cells. Thus, the plant extract may have led to a regeneration of the surviving beta cells (Ojo O. A. et al., 2020). Higher HOMA-IR and lower HOMA-β scores in the diabetic control group indicate the induction of partial pancreatic beta-cell dysfunction and insulin resistance, which confirms the diabetic condition as previously noted elsewhere (Ojo et al., 2017). The reversal of the HOMA-IR and HOMA-β scores following treatment with the extract may, therefore, indicate decreased insulin resistance and restoration or regeneration of the hepatic beta cells.

Oxidative stress is linked to DM through free radicals. Free radical generation accompanied by antioxidant defense impairment could lead to the oxidation of glucose, glycation of protein, and oxidative degradation of protein glycation (Balogun and Ashafa 2017; Yusuf et al., 2021). DM induction by alloxan is said to be through the generation of ROS, leading to rapid destruction of the beta cells of the pancreas, causing hyperglycemia (Yusuf et al., 2021). Elevated pancreatic and hepatic MDA levels after alloxan administration indicate that oxidative stress has occurred because of a compromised antioxidant system in the diabetic condition (Ojo OA. et al., 2020). The reversal of this oxidative damage-prone condition with a reduction of the MDA level with administration of either metformin or AECPS may imply an improved antioxidant status. Thus, our findings may affirm the antioxidant and antidiabetic potential of the extract. Also, decreases in pancreatic and hepatic antioxidant (CAT, SOD, GPX, GSH, and GST) activities in the diabetic-induced rats reveal that oxidative stress resulted from alloxan administration. An increase in enzyme activity in diabetic rats treated with metformin and AECPS revealed an improved antioxidant status (Balogun and Ashafa 2017).

Cardiovascular complications are a common cause of death in diabetics, and postprandial glucose elevation is a risk factor for cardiovascular disease. One manifestation of cardiovascular disease is alteration of the lipid profile. Studies have associated diabetes with hypertriglyceridemia, which may be caused by insulin deficiency. In the current study, we found an abnormal lipid profile in rats after DM induction. In a normal state, it was reported that insulin activates lipoprotein lipase, which hydrolyzes triglycerides, however, in a diabetic state, the enzyme’s inactivation may result in hypertriglyceridemia (Yusuf et al., 2021). Insulin inhibits HMG-CoA reductase, an enzyme that catalyzes the rate-limiting step in cholesterol synthesis, resulting in hypercholesterolemia (Ojo O. A. et al., 2020). In this current study, diabetes induced by alloxan may have inhibited lipid metabolism, as evidenced by elevated TC, TG, VLDL-c, LDL-c, AI, and CRI levels and by decreased HDL-c levels. Another possible explanation for the observed hyperlipidemia is excessive fat mobilization from adipose tissue as a result of glucose underutilization (Ojo et al., 2017). The high levels of CRI in the diabetic control group indicate a proclivity for coronary disease (Ojo et al., 2017). The administration of metformin or the extract to the diabetic rats could have reduced the levels of these lipids with the extract performing better than the standard drug in reducing TG, VLDL-c, and LDL-c, lending credence to the antidiabetic activity of the AECPS. AECPS at 16.8 mg/kg performed better overall than the lower doses of extract administered.

Hexokinase, a vital enzyme for the regulatory step in glycolysis was reduced in the diabetic rat’s relative to the treatment groups. This finding correlates with results reported by Balogun and Ashafa, 2017 that showed a reduced activity of hexokinase attributable in part to declining insulin levels and a decreased mRNA expression of hexokinase in a diabetic state. The improved hexokinase activity by AECPS may facilitate glucose utilization for ATP production (Balogun and Ashafa, 2017).

Glucose-6-phosphatase (G-6-Pase) is a crucial enzyme in maintaining blood glucose homeostasis and is vital during hypoglycemia as it replenishes the blood glucose level. A depletion in the activity of G-6-Pase results in a metabolic disruption identified by hypoglycemic activity activated by cAMP and inhibited by insulin. Insufficient insulin activity in DM elevates G-6-Pase activity leading to elevated blood glucose levels. The result obtained in this study showed that either AECPS or metformin administration diminished G-6-Pase activity in alloxan-induced diabetic rats. The decline in G-6-Pase activity may indicate a decrease in gluconeogenesis and glucose production (Murali et al., 2013).

Fructose-1,6-bisphosphatase (F-1,6-BPase), a gluconeogenic enzyme, is essential in aiding glucose release for circulation in a diabetic state. We showed that its activity increased in diabetic rats because of insufficient insulin. Both AECPS and metformin treatment facilitated a reduction in the levels of F-1,6-BPase in diabetic rats. The activities of F-1,6-BPase enzyme as altered by AECPS or metformin could either occur because of suppression of gluconeogenic and glycolytic activities or modulation by activating metabolism (Balogun and Ashafa 2017; Yusuf et al., 2021).

**FIGURE 4:**
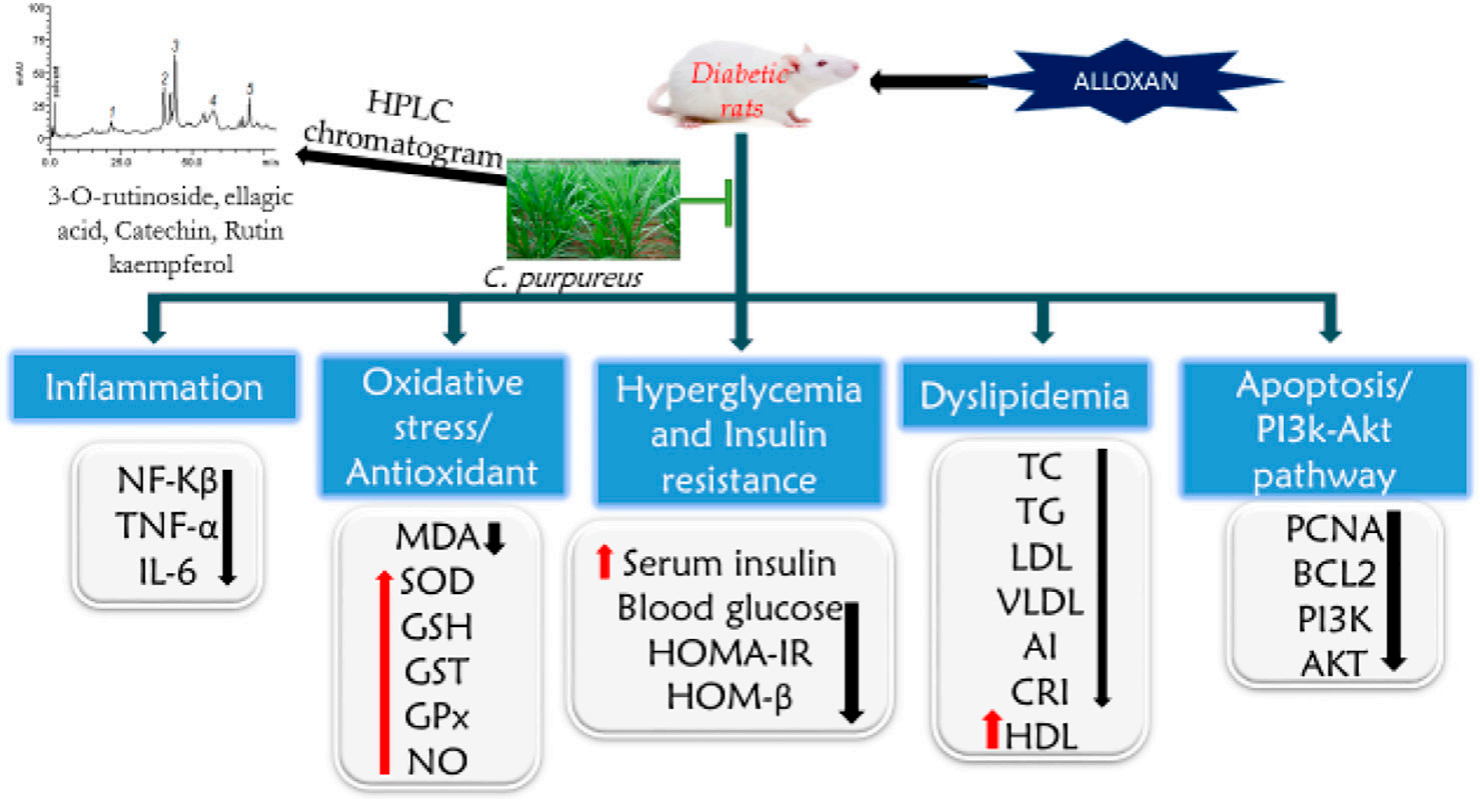
Proposed mechanism of action of *Cenchrus purpureus* shoots in diabetic rats on improving insulin binding and increase glucose metabolism. *C. purpureus*, increase the PI3K/Akt, Bcl2, and PCNA expression in the insulin signaling pathways. This leads to increase in the insulin sensitivity and reduce the blood glucose.

PI3K and AKT are major players in the insulin signaling pathway in DM (Deng et al., 2018). The activation of the PI3K/AKT pathway inhibits increased blood glucose-induced apoptosis in cells (Cheng et al., 2013). The action of insulin is facilitated by the activation of PI3K and its effectors, the protein kinase B (PKB/AKT) kinases. The AMPK signaling pathway increases the impact of the insulin-independent response for glucose uptake. This study revealed that AECPS upregulates the mRNA expression of PI3K and AKT, lowering the FBG level, enhancing insulin levels, and protecting the liver and pancreas. AECPS may protect against alloxan-induced damage through an anti-apoptotic effect via raising the expression of phosphorylation of AKT (Cheng et al., 2013). AECPS upturned the reduced mRNA expression of PI3K and AKT levels in diabetic rats (Figure 4).

The anti-apoptotic Bcl-2 is a major molecule implicated in apoptosis and related to liver and pancreatic damage (Yao et al., 2017). The alloxan-induced diabetic rats had reduced Bcl-2 expression in the liver and pancreas tissues while treatment with AECPS altered the balance of the anti-apoptotic (Bcl-2) molecules and prevented cell death of the hepatic and pancreatic cells at 6.76 and 13.53 mg/kg. We observed a similar effect for the metformin treatment group. The apoptotic pathway, in this case, requires additional examination to determine whether caspase(s) and cytochrome c are involved as documented in other research experiments (Yao et al., 2017).

As a valuable proliferation marker, proliferating cell nuclear antigen (PCNA) expression performs exclusive functions at the onset of cell propagation by facilitating DNA polymerase. PCNA also performs key functions in the eukaryotic cell cycle and facilitates the formation of antibodies to foreign compounds (Omotuyi et al., 2018). In this study, the mRNA expression of PCNA was up-regulated in hepatocytes and pancreatic tissues of the normal rats and downregulated in the diabetic rats, and AECPS improved PCNA expression in the liver and pancreatic tissues of diabetic rats.

In summary, this study shows that AECPS could provide relief from diabetic indications in rats via regulating PI3K/AKT signaling and fatty acid metabolism. The findings from this study showed that AECPS could promote the breakdown of fatty acids, lower the blood glucose level, improve diabetic indications, and protect the liver and pancreas by avoiding apoptosis. Hence, this study implies that AECPS could be a promising candidate for developing an efficient hypoglycemic remedy to offer respite from diabetic indications.

## Data Availability

The original contributions presented in the study are included in the article/Supplementary Material, further inquiries can be directed to the corresponding author.

## Abbreviations

AECPS, Aqueous extract of *Cenchrus purpureus* shoots; AKT, Protein kinase B; Bcl-2, B-cell lymphoma two; CAT, Catalase; cDNA, Complementary DNA; DPPH, 2,2-diphenyl-1-picrylhydrazyl; F-1,6-BP, Fructose-1,6-bisphosphatase; FBG, Fasting blood glucose; FRAP, Ferric reducing antioxidant power; FRIN, Forestry Research Institute of Nigeria; GADPH, Glyceraldehyde-3-phosphate dehydrogenase; GAE, Gallic acid; GSH, Glutathione reduced; G6P, Glucose-6-phosphatase; GPx, Glutathione peroxidase; GST, Glutathione-S-transferase; HOMA-IR, Homeostasis model assessment of insulin resistance; HOMA-*β*, Homeostasis model assessment of *ß*-cell score; HPLC, High performance liquid chromatography; IL-6, Interleukin-6; MDA, Malondialdehyde; NF-κB, Nuclear factor kappa B; NO, Nitric oxide; PI3K, Phosphoinositide 3-kinase; PCNA, Proliferating cell nuclear antigen; PCR, Polymerase chain reaction; PPSE, *C. purpureus* shoots extract; RNA, Ribonucleic acid; SOD, Superoxide dismutase; TNF-α, Tumor necrosis factor alpha; QUE, Quercetin.
