# Supplementary material for: Antidiabetic Activity of Elephant Grass (Cenchrus Purpureus (Schumach.) Morrone) via Activation of PI3K/AkT Signaling Pathway, Oxidative Stress Inhibition, and Apoptosis in Wistar Rats
Source: Front Pharmacol. 2022 Mar 2;13:845196. doi: 10.3389/fphar.2022.845196 (PMC8924541; doi:10.3389/fphar.2022.845196)
Supplement: Supplementary file 1 [file Image1.pdf]

Antidiabetic activity of Elephant grass (*Cenchrus purpureus* (Schumach.) Morrone) via activation of PI3K/AkT signaling pathway, oxidative stress inhibition, and apoptosis in Wistar rats

Oluwafemi Adeleke Ojo[1*], Abosede Itunuoluwa Oni[1], Susan Grant[1], Jennifer Amanze[1], Adebola Busola Ojo[2], Odunayo Anthonia Taiwo[3], Rodelwma Fibilis Maimako[1], Ikponmwosa Owen Evbuomwan[4], Matthew Iyobhebhe[1], Charles Obiora Nwonuma[1], Omorefosa Osemwegie[4], Anthonia Oluyemi Agboola[5], Christopher Akintayo[6] Nnaemeka Tobechukwu Asogwa[7], Nada H. Aljarba[8], Saad Alkahtani[9], Gomaa Mostafa-Hedeab[10] Gaber El-Saber Batiha[11], Oluyomi Stephen Adeyemi[1]

[1] Department of Biochemistry, Landmark University, PMB 1001, Omu-Aran, Nigeria
[2] Department of Biochemistry, Ekiti State University, Ado-Ekiti, Nigeria
[3] Department of Biochemistry, Chrisland University, Abeokuta, Nigeria
[4] Department of Microbiology, Landmark University, PMB 1001, Omu-Aran, Nigeria
[5] Department of Biochemistry, Wesley University, Ondo, Nigeria
[6] Department of Physiology, Afe Babalola University, Ado-Ekiti, Nigeria
[7] Central Research Laboratory 123B, University Road, Tanke Ilorin, Nigeria
[8] Department of Biology, College of Science, Princess Nourah bint Abdulrahman University, Riyadh 11671, Saudi Arabia
[9] Department of Zoology, College of Science, King Saud University, Riyadh, Saudi Arabia
[10] Department of Pharmacology and Health Research Unit, Jouf University, Saudi Arabia
[11] Department of Pharmacology and Therapeutics, Faculty of Veterinary Medicine, Damanhour University, Damanhour, AlBeheira 22511, Egypt.

[*]Corresponding author address: oluwafemiadeleke08@gmail.com; Tel: +2347037824647

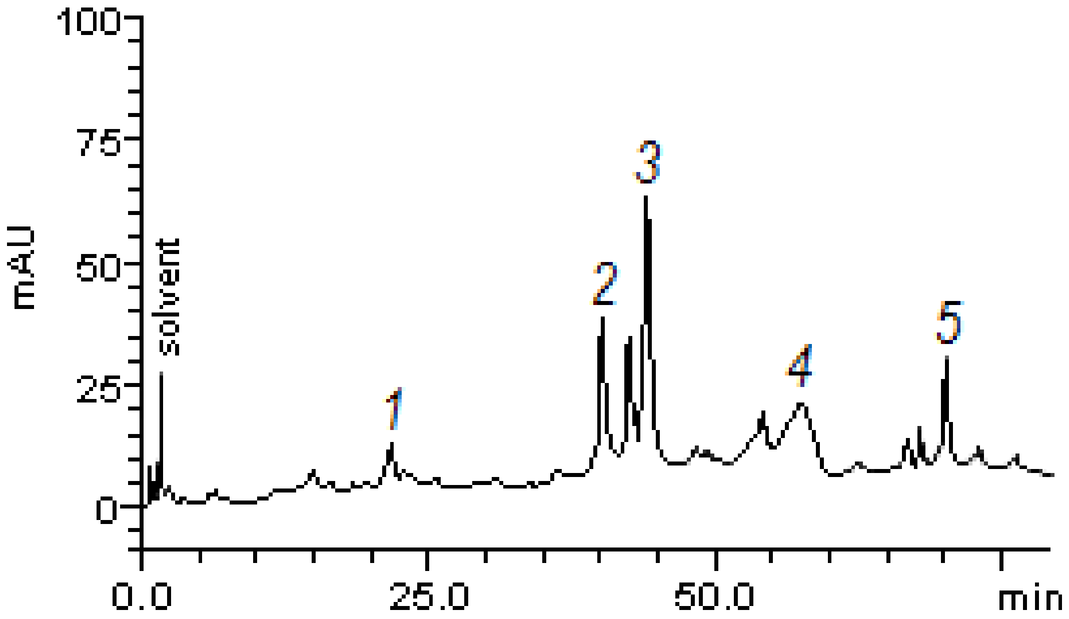

Figure S1: HPLC chromatogram of AECPS. Rutin (peak 1), ellagic acid (peak 2), 3-O-rutinoside (peak 3), catechin (peak 4) and kaempferol (peak 5).

Legend: AECPS: aqueous extract of *Cenchrus purpureus* shoots
